# Estimation of nitrate nitrogen content in cotton petioles under drip irrigation based on wavelet neural network approach using spectral indices

**DOI:** 10.1186/s13007-021-00790-x

**Published:** 2021-08-18

**Authors:** Zhiqiang Dong, Yang Liu, Baoxia Ci, Ming Wen, Minghua Li, Xi Lu, Xiaokang Feng, Shuai Wen, Fuyu Ma

**Affiliations:** 1 School of Agriculture, Shihezi University, Shihezi, 832003 Xinjiang People’s Republic of China; 2 National and Local Joint Engineering Research Center of Information Management and Application Technology for Modern Agricultural Production (XPCC), Shihezi, 832003 People’s Republic of China

**Keywords:** Nitrate nitrogen, Cotton, Petiole, Remote sensing, Wavelet neural network

## Abstract

**Background:**

Estimation of nitrate nitrogen (NO_3_^−^–N) content in petioles is one of the key approaches for monitoring nitrogen (N) nutrition in crops. Rapid, non-destructive, and accurate evaluation of NO_3_^−^–N contents in cotton petioles under drip irrigation is of great significance.

**Methods:**

In this study, we discussed the use of hyperspectral data to estimate NO_3_^−^–N contents in cotton petioles under drip irrigation at different N treatments and growth stages. The correlations among trilateral parameters and six vegetation indices and petiole NO_3_^−^–N contents were first investigated, after which a traditional regression model for petioles NO_3_^−^–N content was established. A wavelet neural network (WNN) model for estimating petiole NO_3_^−^–N content was also established. In addition, the performance of WNN was compared to those of random forest (RF), radial basis function neural network (RBF) and back propagation neural network (BP).

**Results:**

Between the blue edge amplitude (Db) and blue edge area (SDb) of the blue edge parameters was the optimal index for the estimation model of petiole NO_3_^−^–N content. We found that the prediction results of the blue edge parameters and WNN were 7.3% higher than the coefficient of determination (R^2^) of the first derivative vegetation index and WNN. Root mean square error (RMSE) and mean absolute error (MAE) were 25.2% and 30.9% lower than first derivative vegetation, respectively, and the performance was better than that of RF, RBF and BP.

**Conclusions:**

An inexpensive approach consisting of the WNN algorithm and blue edge parameters can be used to enhance the accuracy of NO_3_^−^–N content estimation in cotton petioles under drip irrigation.

## Background

Optimal management of nitrogen (N) fertilizer is important to in the improvement of cotton yield and quality [1], as well as in the reduction of waste and environmental problems associated with excess N fertilizer input [2]. A reasonable amount of N fertilizer is conducive for the balance between vegetative growth in cotton, and promotion of N absorption as well as utilization [3, 4]. The N fertilizer is generally stored and assimilated by cotton plants in the form of nitrate nitrogen (NO_3_^−^–N). NO_3_^−^–N contents vary in different parts of the cotton plant in the order of petioles > stems > leaves [5, 6]. Therefore, petiole NO_3_^−^–N content is an effective parameter that reflects overall N nutrition status of cotton, and petioles can be used as primary plant parts for diagnosing N nutrition [7–9]. Petioles also facilitate rapid determination of N nutrition status of plants to guide rational N fertilizer application [10, 11].

The traditional methods for evaluating cotton N nutrition include soil mineral N determination, laboratory analysis of the plant and determination of petiole NO_3_^−^–N levels among others [12, 13]. However, these methods are associated with certain limitations such as cumbersome procedures, that are time consuming, poor timing of analyses results, and they also involve destructive sampling of many plants [14, 15]. Due to its non-destructive, cheap, and efficient characteristics, hyperspectral remote sensing technology has been used to estimate physiological parameters during crop growth and development [16]. Diagnosis of N nutrition in crops based on spectral data has made considerable progress [17]. The technique has been applied in several crops to obtain crop N nutrition status spectral indices [18–20]. Based on spectral indices, various crop N nutrition monitoring models have been established, and they have achieved a high accuracy. Abulaiti et al. [21] proposed a novel approach for characterizing the Total Nitrogen Content (TNC) by canopy spectral reflectance through a fractional order derivative (FOD) and optimized spectral indices (NDSI, RSI). Rao et al. [22] confirmed the potential of the EO-1 Hyperion hyperspectral sensor for the estimation of total chlorophyll and nitrogen concentrations in cotton crops by developing regression models between hyperspectral reflectance and laboratory measurements of leaf total chlorophyll and nitrogen concentrations. Studies on hyperspectral estimation of NO_3_^−^–N content are limited and most of which focus on plant N content. Gautam et al. [23] used two neural network architectures (Back Propagation and Radial Basis Function) were used to develop twenty different models to predict corn crop NO_3_^−^–N content. They found that radial basis function model based on green vegetation index textural features provided the best performance with an average accuracy of 92.1%.

In addition, parameters associated with spectral location characteristics, trilateral parameters reflect spectral characteristics of vegetation and are also sensitive to variations in N content [24]. The red edge parameter, which is one of the trilateral parameters has been used to estimate N nutrition various crops with satisfactory outcomes [25, 26]. The red edge blue shift phenomenon exists in reflectance spectra of numerous crops. Railyan [27] and Gilbert [28] established that the position and red edge slope in triticale and maize constantly varied during the entire growing season, and were closely associated with the phonological period of crops. The red edge shifted to the long wave direction in the vegetative growth stage, and shifted to the short wave direction in the reproductive growth stage.

Spectral indices of crops can be obtained by developing linear or non-linear relationships or by the learning method of artificial neural networks. Spectral indices combined with artificial neural network algorithms have been used to estimate N contents. Based on adaptive differential optimization extreme learning machine, radial basis function (RBF) and particle swarm optimization BP, Feng et al. [29] established quantitative estimation models for N content estimation in rice canopy leaves. To rapidly and accurately estimate N contents in maize in natural environments, Xiu et al. [30] proposed a method for measuring maize N content based on wavelet energy coefficient and back propagation neural network (BP). Compared to the regression analysis model, the method improved the accuracy of corn N content estimation. Wavelet neural network (WNN) [31] is a type of artificial neural network, that is generated by applying wavelet analysis theory to neural network theory. This network exhibits strong non-linear mapping and learning abilities [31]. Current studies on WNN traverse several fields, including medicine [32], industry [33], and finance [34] and has achieved satisfactory results.

Determination of NO_3_^−^–N content in plants during the growing season for N nutrition monitoring is common in Europe [35]. In China, there are related applications in wheat [36] and corn [37], but less in cotton. Few studies have evaluated the efficacy of the hyperspectral technology to monitor NO_3_^−^–N contents in cotton petioles. A combined WNN model, which has strong adaptive and fault tolerant abilities, can effectively estimate the advantages of linear and nonlinear functions, and facilitate the estimation of petiole NO_3_^−^–N contents in cotton under drip irrigation to provide technical references for cotton growth and N nutrition diagnosis under drip irrigation. Therefore, in this study, we selected spectral indices and trilateral parameters that are sensitive to N and used them to estimate the NO_3_^−^–N content of cotton petioles based on experimental cotton conditions such as drip irrigation, and various N application levels in Xinjiang.

## Methods

### Experimental design

**Fig. 1:**
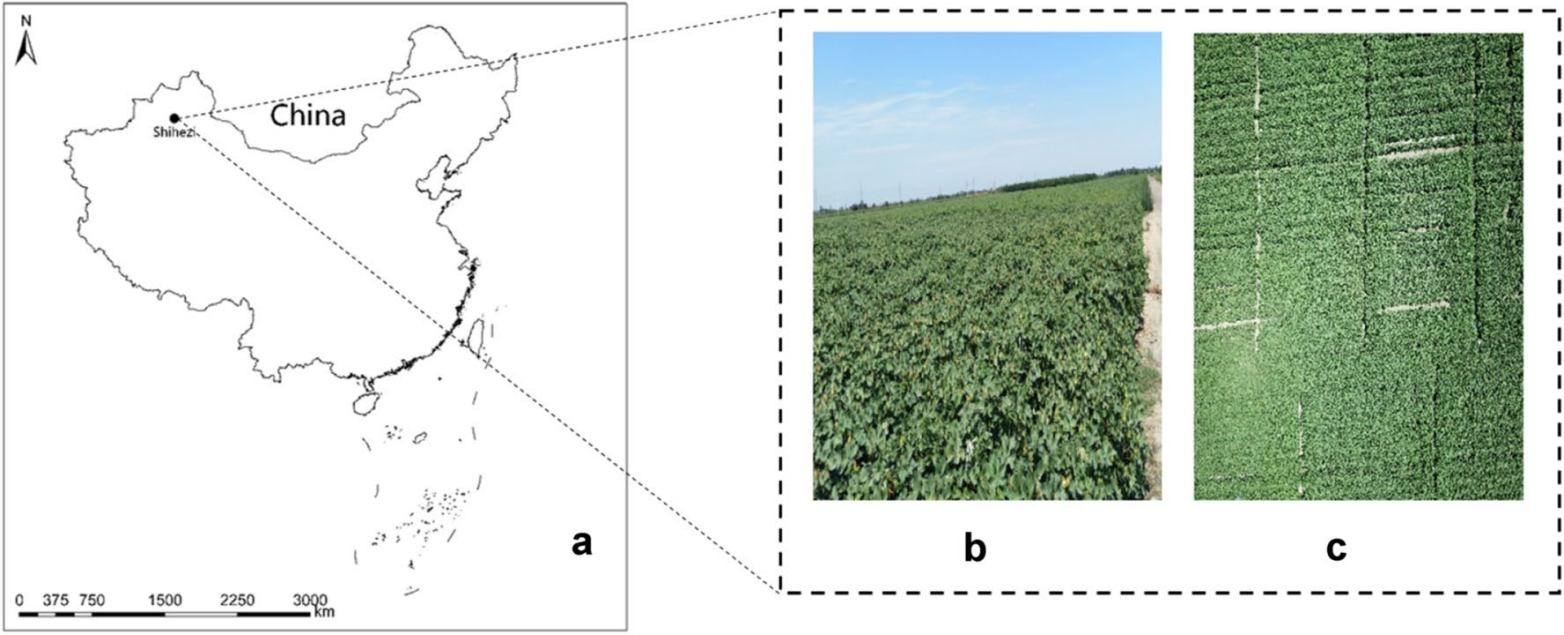
Study area location. **a** Location of the study area; **b** field experiment; **c** high yield verification cotton field photographed by unmanned aerial vehicle (UAV)

The field experiment was performed in 2019 at the teaching experiment farm of Shihezi University, Shihezi City, Xinjiang Uygur Autonomous Region (86° 02′ E, 44° 18′ N) (Fig. 1a, b). Soil fertility of the 0–20 cm soil layer in the experimental plots was determined; total N was 1.13 g/kg, alkali-hydro N was 44.26 mg/kg, available phosphorus content was 19 mg/kg, available potassium content was 486 mg/kg, organic matter content was 15.50 g/kg, while the pH was 8.17. The Lumianyan 24 cotton variety, which is a middle-late maturing variety, with a growth period of approximately 130 days was used as the experiment material. Lumianyan 24 was planted on 23rd April and was harvested on 5th October in 2019.

Five N rates were designed as follows: 0 kg/ha (N0), 195.5 kg/ha (N1), 299 kg/ha (N2), 402.5 kg/ha (N3) and 506 kg/ha (N4). Total amounts of phosphate (P_2_O_5_) and potassium (K_2_O) fertilizers were 109.8 kg/ha and 91.8 kg/ha, respectively. One film, three rows, and three belts were used in the experiment. Row spacing was 76 cm while plant spacing was 10 cm. Each treatment was performed in triplicate and arranged in completely randomized blocks covering a plot area of 2.25 m × 15 m. Cotton was first grown in the experimental field after which protective rows were set around the cotton plants. Other field management measures were in accordance with the requirements of high-yield cultivation. Fertilizers were applied with irrigation water during the cotton growth period under drip irrigation with film.

Validation test data were obtained from a high-yield cotton field in Shihezi university teaching experimental field (Fig. 1c). The independent test cotton field was divided into 15 plots. The total amounts of N fertilizer applied was 300 kg/ha, while total amount of P_2_O_5_ and K_2_O fertilizers applied were 109.8 kg/ha and 91.8 kg/ha, respectively.

### Spectral data acquisition

Key growth periods of cotton were defined as follows: full bud period (65 days after sowing), initial flowering period (77 days after sowing), full flowering period (88 days after sowing), and initial boll stage (107 days after sowing). The analytical spectral devices (ASD) FieldSpec 3 portable spectrometer (Analytical Spectral Devices Inc., Boulder, CO, Colorado, USA) was used to obtain spectral data of the cotton canopy. The band range was 350–1075 nm while the field of view was 25°. Three rows of cotton plants with uniform growth in different treatment plots were randomly selected. The spectrometric probe was vertically placed downward at 25 cm above the canopy. The trigger was pulled during scanning and the obtained spectral data automatically saved. Spectral data acquisition time was three hours. Average values of the three curves were calculated using the Viewspec software (Analytical Spectral Devices, Inc., Boulder, CO, Colorado, USA) as reflectance values of cell spectra.

### Determination of NO_3_^−^–N content in cotton petioles

After the collection of canopy spectral data, 20 cotton plants with petioles (10 days after topping) and two leaves (10 days after topping) were randomly selected from the experimental plots. Cotton petioles and leaves were separated. Petioles were cut and pressed, and the sap was immediately measured using the LAQUA twin NO_3_^−^ meter (HORIBA Inc., Japan), The LAQUA twin NO_3_^−^ meter is a rapid and effective method for evaluating nitrogen levels. The test process, which is widely used in crops nitrogen diagnosis, is simple and accurate [35, 37]. A brief description of NO_3_^−^ meter is presented in Table 1.

**Table 1:** LAQUA twin NO_3_^−^–N instrument profile

Instruments name	LAQUA twin NO_3_^−^	
Measuring principles	Ion electrode	
Volume of samples required	0.3–2.0 mL	
Scope of measurement	2–9900 mg/L	

### Spectral parameter selection

Spectral indices were associated with cotton photosynthesis, soil fertility level, and nutrient management among others. Six spectral indices and trilateral parameters that are sensitive to N nutrition in cotton under drip irrigation were selected based on spectral response characteristics of cotton canopy under drip irrigation and previous studies, as shown in Table 2.

**Table 2:** Calculation methods and reflectance of spectral indices

Spectral index		Abbreviation	Formula	References
Trilateral parameters	Red edge amplitude	Dr	Maximum first derivative within 680–760	[38]
	Red edge area	SDr	Sum of first derivative values in red edge	[38]
	Yellow edge amplitude	Dy	Maximum first derivative within 560–640	[39]
	Yellow edge area	SDy	Sum of first derivative values in yellow edge	[39]
	Blue edge amplitude	Db	Maximum first derivative within 490–530	[39]
	Blue edge area	SDb	Sum of first derivative values in blue edge	[39]
Vegetation index	Red edge ratio spectral index	RD	R_740_/R_720_	[40]
	Red edge model index	CI_red-edge_	(R_780_/R_710_) − 1	[41]
	Normalized difference red edge index	NDRE	(R_790_R_720_)/(R_790_ + R_720_)	[42]
	Normalized difference spectral index	ND705	(R_750_ − R_705_)/(R_750_ + R_705_)	[43]
	Near infrared ratio spectral index	NIR	R_780_/R_740_	[44]
	Red edge ratio spectral index	RI-1 dB	R_735_/R_720_	[45]

WNN was used to establish the estimation model of cotton petiole NO_3_^−^–N contents. Two spectral characteristic indices and two trilateral parameters with strong correlations between critical growth period and petiole NO_3_^−^–N content were selected as independent variables to develop a cotton petiole NO_3_^−^–N content model. Independent validation samples were used to test the regression model. The coefficient of determination (R^2^), root mean square error (RMSE), and mean absolute error (MAE) were used to enhance the accuracy of the model to develop the best estimation model (Eqs. 1–3). Mean relative error (MRE) was used to determine the number of hidden nodes in WNN (Eq. 4).

1 $$ R^{2} = 1 - \frac{{\mathop \sum \nolimits_{i = 1}^{n} \left( {F_{i} - T_{i} } \right)^{2} }}{{\mathop \sum \nolimits_{i = 1}^{n} \left( {T_{i} - \overline{{T_{i} }} } \right)^{2} }} ; $$

2 $$ RMSE = \sqrt {\frac{1}{n} \times \mathop \sum \limits_{i = 1}^{n} \left( {F_{i} - T_{i} } \right)^{2} } ; $$

3 $$ MAE = \frac{1}{n}\mathop \sum \limits_{i = 1}^{n} \left| {F_{i} - T_{i} } \right|; $$

4 $$ MRE = \sqrt {\frac{1}{n} \times \mathop \sum \limits_{i = 1}^{n} \left( {\frac{{F_{i} - T_{i} }}{{T_{i} }}} \right)^{2} } \times 100\% ; $$

whereby, Fi and Ti are the predicted and true values, respectively, while n is the number of samples.

### Modeling methods

WNN [46] is an artificial neural network that is based on wavelet analysis. The S-type activation function of the hidden node in the neural network is replaced with the wavelet function. The corresponding weight from the input layer to the hidden layer, and the threshold value of the hidden layer are replaced with scale expansion and time shift factors of wavelet function, respectively.

Determination of the number of hidden layer nodes is a key factor influencing the accuracy of the WNN prediction model. Therefore, the number of hidden layer nodes is determined under the condition of meeting model accuracy while the compactness of the model structure is ensured to avoid redundancy. In the present study, the number of hidden layer nodes was set to five, and the model was trained with five, eight, 10, 12, 16, and 20 hidden layer nodes. The training error is presented in Table 3. Prediction MRE is considered minimum when the number of hidden nodes is 10. Therefore, the number of hidden nodes was set to 10, the learning rate was 0.01, the number of iterations was 1000, and the maximum allowable error was 0.001. WNN was created using the MATLAB R2019b software (MathWorks, Inc. Natick, Massachusetts, USA).

**Table 3:** Influence of the number of nodes in different hidden layers on network prediction error

Learning rate	Number of iterations	Maximum allowable error	Number of hidden layer nodes	MRE%
0.01	1000	0.001	5	7.14
0.01	1000	0.001	8	5.86
0.01	1000	0.001	10	5.82
0.01	1000	0.001	12	6.92
0.01	1000	0.001	16	6.35
0.01	1000	0.001	20	5.92

**Fig. 2:**
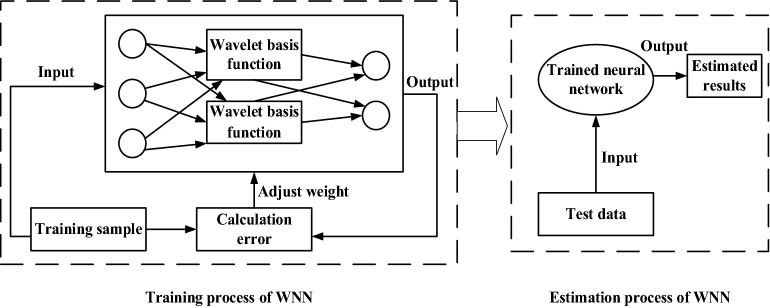
Operation of WNN

According to the WNN theory, through repeated trainings and iteration, a WNN estimation model for petiole NO_3_^−^–N content in cotton under drip irrigation was developed based on spectral indices as shown in Fig. 2.

Random forest (RF) [46] is an algorithm that integrates multiple trees through ensemble learning and its basic unit is a decision tree. RF is commonly used in high-dimensional data classification and regression. The RF algorithm was developed using the MATLAB R2019b software. The number of classification trees in the RF algorithm was 1070.

The RBF neural network [46] can fit continuous nonlinear functions, and its hidden layer adopts RBF, which locally responds to input signals. In this study, the RBF neural network was developed using the MATLAB R2019b software. The variance parameter of RBF kernel function was set to 0.3.

The BP neural network [46] is a learning algorithm of feedback networks, that reflects input–output relationships of samples, and has a strong non-linear fuzzy approximation ability. In this study, a BP neural network was developed using the MATLAB R2019b software. The BP neural network adopts a three-layer structure, with 10 hidden layer nodes, 1000 iterations, and 0.01 learning rate.

## Results

### The relationship between NO_3_^−^–N contents in petioles and trilateral parameters

Table 4 shows that the correlation between NO_3_^−^–N content in petioles and blue edge parameters was stronger than that of red edge and yellow edge parameters. Although the correlations were significant; a negative correlation was observed among red edge amplitude (Dr), red edge area (SDr), yellow edge amplitude (Dy), and yellow edge area (SDy), while a positive correlation was observed between blue edge amplitude (Db) and blue edge area (SDb). Correlation coefficient of Db and SDb was 0.90.

**Table 4:** Correlation coefficient between NO_3_^−^–N content of petioles and trilateral parameters

Trilateral parameters	Correlation coefficients
Dr	− 0.80**
SDr	− 0.72**
Dy	− 0.85**
SDy	− 0.59**
Db	0.90**
SDb	0.90**

**Means significant at the level of *p* < 0.01

From the regression analysis, Table 5 shows that blue edge parameters and petiole NO_3_^−^–N content R^2^ was higher while RMSE was lower when compared to those of red edge and yellow edge parameters. The polynomial regression equation of Db exhibited the highest coefficient of determination (R^2^ = 0.89), while the RMSE value of Db linear regression equation was the lowest (RMSE = 1.04 g/L). Based on linear regression equations of blue edge, red edge and yellow edge parameters, the R^2^ value of Db in blue edge parameters increased by 25.0% and 11.1%, respectively, when compared to Dr in red edge parameters and Dy in yellow edge parameters. Moreover, the R^2^ value of SDb in blue edge parameters increased by 55.8% and 11.0%, respectively, when compared to SDr in red edge parameters and SDy in yellow edge parameters.

**Table 5:** Quantitative relationship between trilateral parameters and NO_3_^−^–N content in petioles

Trilateral parameters	Functional model	Regression equation	R^2^	RMSE (g/L)
Dr	Linear	y = − 1E + 06x + 21,084	0.64	1.40
	Exponential	y = 59,355e^−160.9x^	0.61	1.45
	Quadratic	y = − 2E + 08x^2^ + 6E + 06x − 23990	0.67	2.93
SDr	Linear	y = − 12,321x + 13,673	0.52	2.28
	Exponential	y = 18,355e^−1.876x^	0.47	2.59
	Quadratic	y = − 58,926x^2^ + 48,072x − 895.38	0.60	2.53
Dy	Linear	y = − 1E + 08x + 1114.1	0.72	1.08
	Exponential	y = 2691.4e^−17931x^	0.67	1.82
	Quadratic	y = − 4E + 12x^2^ − 5E + 08x − 9212.4	0.84	2.73
SDy	Linear	y = − 193,244x − 1139.4	0.73	2.91
	Exponential	y = 1924.3e^−29.42x^	0.67	2.54
	Quadratic	y = − 8E + 06x^2^ − 905,730x − 16273	0.79	2.51
Db	Linear	y = 4E + 06x − 836.47	0.80	1.04
	Exponential	y = 2036.5e^619.48x^	0.72	1.50
	Quadratic	y = − 3E + 09x^2^ + 2E + 07x − 14359	0.89	1.64
SDb	Linear	y = 498,071x − 9405.9	0.81	1.45
	Exponential	y = 492.37e^78.986x^	0.80	1.53
	Quadratic	y = 9E + 06x^2^ − 84,605x − 199.84	0.82	1.98

E stands for scientific counting; e is the base of natural logarithm

### Relationship between NO_3_^−^–N content in petioles and vegetation indices

Correlation analysis (Table 6) revealed a significant negative correlation between vegetation index red edge (RD) and NO_3_^−^–N content in petioles, with a correlation coefficient of − 0.81, followed by near infrared ratio spectral index (NIR), with a correlation coefficient of − 0.79, and other vegetation indices that reached significant correlation levels (Table 6). Correlation analyses of NO_3_^−^–N content in cotton petioles under drip irrigation and vegetation indices developed based on the first six sets of derivative spectral data revealed that ND705 was significantly negatively correlated with NO_3_^−^–N content in petioles, and the correlation coefficient was − 0.90, followed by red edge model index (CI_red-edge_), with a correlation coefficient of − 0.89 (Table 6). Correlation coefficients of ND705 and NO_3_^−^–N contents in petioles were significantly increased by 18.4% and 20.3%, respectively except for NIR based on ND705 and CI_red-edge_.

**Table 6:** Correlation coefficient between NO_3_^−^–N content of petioles and vegetation indices

Type of reflectivity	Vegetation indices	Correlation coefficients
First derivative	RD	− 0.85**
	CI_red-edge_	− 0.89**
	NDRE	− 0.80**
	ND705	− 0.90**
	NIR	− 0.62**
	RI-1 dB	− 0.72**
Original	RD	− 0.81**
	CI_red-edge_	− 0.74**
	NDRE	− 0.77**
	ND705	− 0.76**
	NIR	− 0.79**
	RI-1 dB	− 0.77**

**Means significant at the level of *p* < 0.01

Regression analyses (Table 7) between NO_3_^−^–N contents in petioles and six vegetation indices during the key growth period of cotton under drip irrigation revealed that R^2^ values of the first derivative vegetation indices, RD, CI_red-edge_, normalized difference red edge index (NDRE), and normalized difference spectral index (ND705) were higher than those of the original vegetation indices, and that RMSE values were lower than those of original vegetation indices. Among the three regression models, the R^2^ value of the polynomial regression equation for the first derivative vegetation index, ND705 was the highest (R^2^ = 0.83), while the linear regression equation of the first derivative vegetation index, CI_red-edge_ had the lowest RMSE (0.92 g/L). In conclusion, the first derivative vegetation index, ND705 and CI_red-edge_ exhibited a higher predictive ability. The R^2^ value of the polynomial regression equation between ND705 and petiole NO_3_^−^–N content was 53.4% higher than that of ND705. The RMSE value of the linear regression equation of the first derivative vegetation index and petiole NO_3_^−^–N content was 45.6% lower than that of the original vegetation index, CI_red-edge_.

**Table 7:** Quantitative relationship between spectral indices and NO_3_^−^–N content

Type of reflectivity	Vegetation indices	Functional model	Regression equation	R^2^	RMSE (g/L)
First derivative	RD	Linear	y = − 8116.9x + 13,150	0.71	1.77
		Exponential	y = 17,860e^−1.306x^	0.73	2.01
		Quadratic	y = 3178.2x^2^ − 13,332x + 15,120	0.72	1.88
	CI_red-edge_	Linear	y = − 76,288x − 66,089	0.80	0.92
		Exponential	y = 0.067e^−12.01x^	0.78	0.98
		Quadratic	y = − 169,389x^2^ − 400,646x − 221,261	0.80	0.93
	NDRE	Linear	y = − 27,424x − 18,368	0.63	1.14
		Exponential	y = 147.75e^−4.115x^	0.56	1.23
		Quadratic	y = 126,872x^2^ + 207,536x + 89,944	0.67	1.18
	ND705	Linear	y = − 18,527x + 3265	0.82	1.52
		Exponential	y = 3707.4e^−2.893x^	0.78	1.35
		Quadratic	y = − 29,935x^2^ − 30,687x + 2342.8	0.83	1.69
	NIR	Linear	y = − 85,125x + 10,408	0.39	1.45
		Exponential	y = 11,137e^−12.9x^	0.35	1.55
		Quadratic	y = − 2E + 06x^2^ + 47,910x + 8644.1	0.44	1.44
	RI-1 dB	Linear	y = − 19,373x + 22,583	0.52	2.37
		Exponential	y = 78,853e^−3.076x^	0.52	2.91
		Quadratic	y = 126,872x^2^ + 207,536x + 89,944	0.59	2.83
Original	RD	Linear	y = − 8564.7x + 25,826	0.65	1.50
		Exponential	y = 131,902e^−1.36x^	0.65	1.57
		Quadratic	y = − 10,788x^2^ + 39,287x − 26,804	0.67	1.47
	CI_red-edge_	Linear	y = − 3415.6x + 14,222	0.54	1.69
		Exponential	y = 19,774e^−0.516x^	0.49	1.81
		Quadratic	y = − 357.75x^2^ − 1914.2x + 12,720	0.54	1.67
	NDRE	Linear	y = − 28,351x + 17,684	0.59	1.70
		Exponential	y = 34,790e^−4.394x^	0.56	1.81
		Quadratic	y = − 202,849x^2^ + 124,311x − 10,381	0.63	1.60
	ND705	Linear	y = − 2745.6x + 16,233	0.58	2.38
		Exponential	y = 29,232e^−0.441x^	0.59	2.41
		Quadratic	y = − 35.761x^2^ − 2493x + 15,800	0.58	2.34
	NIR	Linear	y = − 28,744x + 38,765	0.62	1.66
		Exponential	y = 946,939e^−4.488x^	0.60	1.81
		Quadratic	y = − 180,524x^2^ + 369,826x − 180,634	0.66	1.53
	RI-1 dB	Linear	y = − 10,960x + 28,560	0.59	1.58
		Exponential	y = 199,238e^−1.729x^	0.58	1.67
		Quadratic	y = − 26,273x^2^ + 92,468x − 72,663	0.64	1.52

E stands for scientific counting; e is the base of natural logarithm

### Development and verification of the estimation models

In this study, we used two stable and representative first derivative vegetation indices (ND705, CI_red-edge_) and Db as well as SDb (blue edge parameters) to develop the petiole NO_3_^−^–N content estimation model WNN. Simulated and measured values were fitted and analyzed using an independent validation test data. Results are presented in Table 8. The R^2^, RMSE, and MAE values of the WNN estimation model based on first derivative vegetation indices were 0.81, 0.91 g/L, and 0.73 g/L, respectively, while the R^2^, RMSE, and MAE values of the validation model were 0.82, 0.87 g/L, and 0.68 g/L, respectively. The R^2^, RMSE, and MAE values of the WNN estimation model based on blue edge parameters were 0.88, 0.74 g/L and 0.58 g/L, respectively. The R^2^, RMSE, and MAE values of the validation model based on blue edge parameters were 0.88, 0.65 g/L, and 0.47 g/L, respectively. The R^2^ value of WNN based on blue edge parameters was increased by 8.6%, whereas RMSE and MAE values were reduced by 18.7% and 20.5%, respectively, when compared to the estimation model based on first derivative vegetation indices. The R^2^ value of the validation model based on blue edge parameters was increased by 7.3%, whereas RMSE and MAE values were reduced by 25.2% and 30.9%, respectively, when compared to the estimation model based on first derivative vegetation indices. Generally, the R^2^ value of the validation model was higher than that of the estimation model, while RMSE and MAE values were lower than those of the estimation model, implying that the validation model is stable.

**Table 8:** Modeling and validation of NO_3_^−^–N content in petioles by wavelet neural network

Spectral index type	Modeling				Validation			
	Number of samples	R^2^	RSME (g/L)	MAE (g/L)	Number of samples	R^2^	RSME (g/L)	MAE (g/L)
Vegetation indices	60	0.81	0.91	0.73	60	0.82	0.87	0.68
Blue edge parameters	60	0.88	0.74	0.58	60	0.88	0.65	0.47

The verification model of NO_3_^−^–N content in petioles was developed based on WNN, RF, RBF and BP (Table 9). The R^2^, RMSE and MAE values of the WNN validation model based on first derivative vegetation indices were 0.82, 0.87 g/L, and 0.68 g/L. The R^2^ values of WNN, RF, and RBF were all 0.82. However, the RMSE and MAE values of WNN were 5.4% and 10.5% lower than those of RF, 1.0% and 5.6% lower than those of RBF, and 17.2% and 16.0% lower than those of BP, respectively. The R^2^, RMSE, and MAE values of the WNN validation model based on blue edge parameters were 0.88, 0.65 g/L, and 0.47 g/L, respectively. Compared to RF, the R^2^ value of WNN was increased by 7.3%, whereas RMSE and MAE values were decreased by 17.7% and 21.7%, respectively. Compared to RBF, the R^2^ of WNN was increased by 8.6%, whereas RMSE and MAE values were decreased by 18.8% and 23.0%, respectively. Compared to BP, the R^2^ of WNN was increased by 14.3%, whereas RMSE and MAE values were decreased by 27.0% and 27.7%, respectively.

**Table 9:** Validation between predicted and measured of NO_3_^−^–N content in petioles based on different methods

Methods	Blue edge parameters				Vegetation indices			
	Number of samples	R^2^	RSME (g/L)	MAE (g/L)	Number of samples	R^2^	RSME (g/L)	MAE (g/L)
WNN	60	0.88	0.65	0.47	60	0.82	0.87	0.68
RF	60	0.82	0.79	0.60	60	0.82	0.92	0.76
RBF	60	0.81	0.80	0.61	60	0.82	0.88	0.72
BP	60	0.77	0.89	0.65	60	0.74	1.02	0.81

The R^2^ value of the WNN, based on blue edge parameters, increased by 7.3% while RMSE and MAE values reduced by 25.2% and 30.9%, respectively, when compared to the model based on first derivative vegetation indices. The RMSE and MAE values of the RF based on blue edge parameter values were reduced by 14.1% and 21.1%, respectively, when compared to the model based on first derivative vegetation indices. The RMSE and MAE values of the RBF based on blue edge parameter values were reduced by 9.1% and 15.3%, respectively, when compared to the model based on first derivative vegetation indices. The R^2^ value of BP based on blue edge parameters was increased by 4.1% while RMSE and MAE values were reduced by 12.7% and 19.8%, respectively, when compared to the model based on first derivative vegetation indices.

## Discussion

### Feasibility of remote sensing monitoring NO_3_^−^–N content in cotton petioles under drip irrigation

Timely and accurate monitoring of N contents in crops is key to accurate application of N fertilizer [47]. Advances in remote sensing technology present a potential novel method for monitoring crop nutrition [48]. This technology has been used to monitor plant N contents and N accumulation, although studies on NO_3_^−^–N contents in cotton petioles under drip irrigation are scarce [49]. Monitoring of petiole NO_3_^−^–N contents is widely used to evaluate crop nutrition and to inform top-dressing [48]. In this study, the correlations among six trilateral parameters, six vegetation indices, and NO_3_^−^–N contents in cotton petioles under drip irrigation was revealed that a large proportion of the spectral index was strongly correlated with NO_3_^−^–N content in petioles. Among them, correlation coefficients of Db, SDb, and first derivative ND705 are all 0.90. These findings imply that estimation of NO_3_^−^–N contents in cotton petioles under drip irrigation using spectral indices is feasible.

### Potential of blue edge parameters for the estimation of NO_3_^−^–N content in petioles

Trilateral parameters, especially red edge parameters, effectively reflect the characteristics of crop N status [49, 50]. Studies on wheat, rice, and other crops developed N content estimation models based on red edge parameters, which achieved satisfactory results [51, 52]. In this study, we established that the correlation between blue edge parameters and petiole NO_3_^−^–N contents was strong, and that the traditional regression model of blue edge parameters and petiole NO_3_^−^–N content was superior to the red edge and yellow edge parameters. Estimation and validation models based on blue edge parameters and WNN exhibited a superior capacity to the vegetation index model based on the red edge band. This finding is relatively inconsistent with the findings of most previous studies, which focused on the correlation between red edge parameters and crop N. Blue edge is sensitive to crop N. Li et al. [53] determined N levels in winter wheat by performing hyperspectral analyses, and established that blue-violet light was sensitive to N. Stroppiana et al. [54] reported that the blue light is the ideal wave segment for N estimation in rice. Our findings could be attributed to variations in crop canopy structure and biomass, the unique climatic conditions in Xinjiang, drip irrigation fertilization methods, or to other factors.

When selecting spectral characteristic parameters, most of the studies have evaluated red edge parameters and paid less attention to the blue edge parameters, which cover wavelengths between 490 and 530 nm. Therefore, blue edge parameters should be considered when determining N levels based on spectral data. We also showed the potential of blue edge parameters in estimating of N levels in crops.

### Applications of neural networks in remote sensing monitoring

The R^2^ value of the WNN estimation and verification models were relatively high, while RMSE and MAE values were relatively low as shown in Tables 8, 9, implying that stability of the model is high while its, estimation capacity is superior. Combined WNN maintains the advantages of artificial neural networks and wavelet analysis, which accelerates network convergence, thereby preventing the algorithm from falling into local optimum and occasionally making local analysis more frequent [55, 56]. The RBF neural network [57] algorithm confers the advantages of rapid training and convergence speed, strong input–output mapping ability, and strong generalization ability when compared to the BP neural network algorithm. Furthermore, our findings confirmed that the estimation model based on the RBF neural network is superior to that of the BP neural network model.

Neural networks [58, 59] exhibit a great potential in learning and developing non-linear complex relationship models, and they exhibit high tolerance for input objects. Wang et al. [60] constructed the Chinese cabbage population quality BP neural network model which effectively monitors, N utilization of Chinese cabbage is monitored effectively. The constructed population quality evaluation model has a high R^2^ value and a comparatively low RMSE value for quality evaluation of Chinese cabbage in different periods. Sabzi et al. [61] used the hyperspectral imaging technology combined with artificial neural networks and imperialist competition algorithm (ANN-ICA) to detect early excessive N levels in cucumber leaves. They found that hyperspectral imaging technology combined with artificial neural networks can detect excess N in plants in near infrared band (NIR), and the correct classification rate is 96.11%. Neural networks can better simulate heteroscedasticity and have the ability to learn hidden relations in data without imposing any fixed relations [57, 64].

**Fig. 3:**
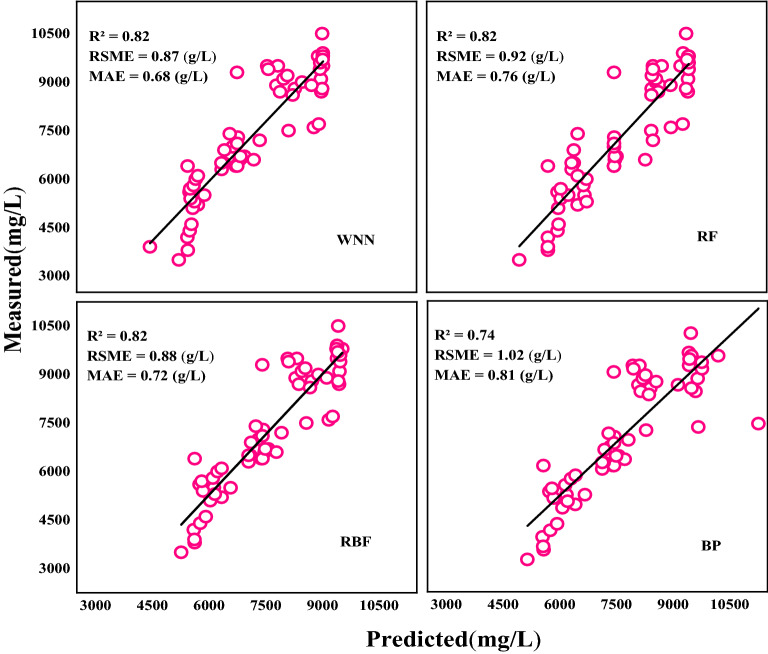
Validation of predicted and measured values of NO_3_^−^–N content in petioles by vegetation indices

**Fig. 4:**
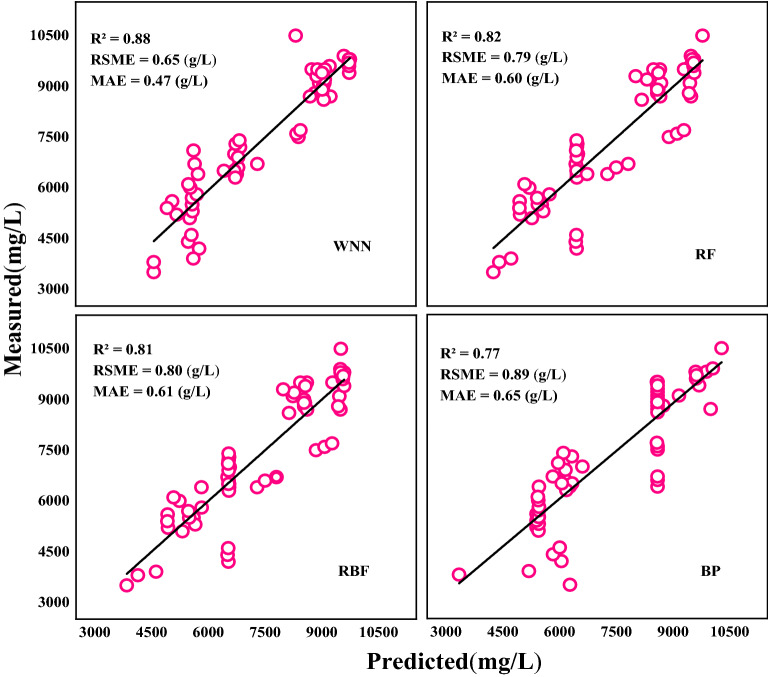
Validation of predicted and measured values of NO_3_^−^–N content in petioles by blue edge parameters

Studies on crop physiological parameter estimation have shown that the RF algorithm exhibits a high accuracy and estimation ability, and confers the advantages of strong stability and high efficiency when compared with other modeling methods. Loozen et al. [62] used the RF technology estimate the N content of a European forest canopy, which exhibited a superior accuracy (R^2^ = 0.62, RMSE = 0.18). To establish an efficient method for estimating winter wheat biomass, Yue et al. [63] used RF algorithm to develop a regression model of winter wheat biomass by combining spectrum, radar backscattering, vegetation index, and radar vegetation index, they found that the stochastic forest algorithm can be applied in remote sensing to estimate the winter wheat biomass. The RF regression algorithm has been shown to result in over fitting and higher test errors when compared to the neural network algorithm [64, 65]. RMSE and MAE values of WNN and RBF models based on the vegetation indices were found to be lower than those of the RF during model validation (Fig. 3). The R^2^ value of WNN based on blue edge parameters was higher than that of the RF, while RMSE and MAE values were lower than those of the RF (Fig. 4), consistent with previous findings that the RF method exhibits a weak predictive ability.

## Conclusions

We analyzed and compared the performance of trilateral parameters and vegetation indices in estimating NO_3_^−^–N contents in cotton petioles under drip irrigation, in addition to determining an effective method for estimating NO_3_^−^–N contents in cotton petioles under drip irrigation using blue edge parameters and WNN. It was found that the correlation between blue edge parameters and petiole NO_3_^−^–N content was 0.90, and the regression equation of blue edge parameters and petiole NO_3_^−^–N content had a higher R^2^ and a lower RMSE. The validation model, which was based on blue edge parameters and WNN, exhibited the highest coefficient (R^2^ = 0.88), lowest root mean square error (RMSE = 0.65 g/L) and lowest mean absolute error (MAE = 0.47 g/L). Therefore, blue edge parameters and WNN can be used to estimate NO_3_^−^–N contents in cotton petioles under drip irrigation.

## Data Availability

The remotely sensed and field sampling data used in this study is available from the corresponding author on reasonable request.
